# Local environments, accessibility and affordability: A qualitative analysis of alcohol purchasing across different socio‐economic areas in Victoria, Australia

**DOI:** 10.1111/dar.14018

**Published:** 2025-02-06

**Authors:** Gabriel Caluzzi, Klaudia Kepa, Alexandra Torney, Michael Livingston, Yvette Mojica‐Perez, Nicholas Taylor, Sarah Callinan, Amy Pennay

**Affiliations:** ^1^ Centre for Alcohol Policy Research La Trobe University Melbourne Australia; ^2^ National Drug Research Institute Curtin University Melbourne Australia

**Keywords:** alcohol, ecological, purchasing, qualitative, socio‐economic status

## Abstract

**Introduction:**

Alcohol purchasing practices—linked to consumption practices and potential harms—vary across socio‐economic environments. While there is an epidemiological link between purchasing and harm, little qualitative research unpacks purchasing practices with an eye to socio‐economic status (SES). Drawing on qualitative interviews, this paper takes an ecological approach to examine how people in different SES areas discuss their alcohol purchasing practices to understand how SES influences alcohol purchasing.

**Methods:**

This analysis draws on 45 interviews with people who drink alcohol in Victoria, Australia conducted in 2023–2024. Participants were purposively recruited from low, middle and high SES neighbourhoods. All interviews were recorded, transcribed and analysed thematically.

**Results:**

Accessibility to alcohol outlets influenced purchasing patterns, with those in low SES neighbourhoods facing barriers to accessing on‐premises venues. Neighbourhood‐level norms shaped feelings of safety and enthusiasm for attending on‐premises venues, often tied to local gentrification. Low SES neighbourhood participants were more constrained by on‐premises costs but less conscious of alcohol budgeting, in contrast to the middle and high SES neighbourhood participants, who were more intentional about their budgeting. Participants from all groups noted cost of living concerns influenced alcohol affordability, contrasting this with the continued affordability of cheap wine.

**Discussion and Conclusion:**

This analysis provides important context as to how neighbourhood SES can influence purchasing practices. Notably, the emphasis on off‐premises purchasing was common across the groups, influenced by cost of living pressures and perceived affordability of wine.

## 1 INTRODUCTION

Alcohol purchasing patterns vary across socio‐economic environments, with implications for consumption and harm. Epidemiological research has examined the relationships between alcohol availability, purchasing practices and related harms [1, 2]. For example, total alcohol consumption, drinking patterns and alcohol‐related harms are influenced by alcohol outlet density and opening hours [3]—factors that can vary according to the socio‐economic status (SES) of an area [4]. Similarly, pricing and taxation strategies for addressing alcohol‐related harms through purchasing can disproportionately affect lower SES groups [5]. Indeed, there are multiple ecological and socio‐economic factors that influence alcohol purchasing.

An ecological perspective highlights the various physical, social, economic and subjective elements that contribute to alcohol purchasing [6]. This includes proximity to on and off‐premises outlets; how common socialising with alcohol occurs; relative affordability of alcohol; and perceived accessibility. For example, high density of alcohol outlets in a neighbourhood has been associated with greater purchasing [7, 8]. A New Zealand study by Connor et al. [8] found a positive association between alcohol outlet density and both ‘binge’ drinking and alcohol‐related problems—an association that was independent of neighbourhood SES. However, the distribution of alcohol outlets is not equal across neighbourhoods. In Victoria, Australia, lower SES areas are more likely to have a higher density of off‐premise alcohol outlets, while on‐premise outlets cluster in more advantaged areas [4].

There are other important ecological factors that have been recognised. A qualitative scoping review by Dimova et al. [9] found that proximity to alcohol outlets can normalise drinking and foster permissive environments. Neighbourhoods of differing SES are also often exposed to different forms of marketing and advertising [10]. At an individual level, SES and disposable income can influence purchasing patterns and context (e.g., drinking off‐premises vs. on‐premises, purchasing larger quantities vs. smaller quantities) [11]. There are also individual, social and cultural factors interlinked with neighbourhood SES that can influence alcohol purchasing.

One of the under‐researched areas is how people talk about their preferences and patterns of alcohol purchasing according to their socio‐economic environments. This could enhance our contextual understanding of barriers and enablers in alcohol purchasing and subsequent patterns of drinking and harm. While survey and administrative data help quantify these patterns, little qualitative research has explored purchasing practices with a focus on neighbourhood socio‐economic status.

### 1.1 Alcohol purchasing in Australia

Australia is characterised by densely populated metropolitan cities, which extend out into suburban areas and more sparsely populated regional and rural towns. Higher SES neighbourhoods are typically concentrated in inner‐metropolitan areas, while outer‐metropolitan areas and regional cities generally have lower SES levels, with the lowest levels found in more rural and remote regions [12]. Australia is considered a high‐income country where most people can purchase alcohol in some form [13]. Off‐premises purchases can be made in store at dedicated ‘bottle shops’, which are often co‐located in or near supermarkets. Although alcohol is highly regulated and rarely sold in supermarkets in Australia, supermarkets such as Aldi can sell unchilled alcohol in store in most Australian jurisdictions (including Victoria). There are also methods of online purchasing including home delivery or pickup. Australia's alcohol taxation system varies according to product type and alcohol content, meaning some alcohols (e.g., spirits) are taxed more than others (e.g., cask wines) [14]. Alcohol is also sold on‐premises in bars, pubs, restaurants and nightclubs, which tend to be more expensive due to venue/service costs.

Self‐report surveys suggest that warehouse‐style bottle shops are the most common source of purchase, followed by smaller bottle shops [15]. Lower‐income consumers tend to purchase cheap wine more so than those with middle and higher incomes [15, 16]. There is also evidence that Australians living in the city prefer not to travel far to purchase alcohol, although price discounts can influence this [17]. Furthermore, increasing online purchasing is also driven by factors like cost and convenience [18].

In this study we take an ecological perspective, focusing on neighbourhood SES, to qualitatively explore how people purchase alcohol according to socio‐economic environments. This approach adds context to how people make decisions around their alcohol purchasing and allows us to answer the research question: how does neighbourhood socio‐economic status influence purchasing patterns?

## 2 METHODS

This study draws on 45 interviews with people who drink alcohol in Victoria, Australia, conducted between September 2023 and January 2024. It was part of a larger mixed methods project exploring how drinking patterns, purchasing patterns and experiences of harms differed among Victorians living in different socio‐economic areas. For the qualitative component, potential participants were recruited through an online screening survey advertised on social media. Eligibility criteria included being 18 years or older, residing in Victoria, consuming alcohol at least fortnightly or drinking five or more standard drinks in an occasion in the month prior to completing the survey, and living in one of three (low, middle, high) socio‐economic areas according to the Socio‐Economic Indexes for Areas [12]. The Socio‐Economic Indexes for Areas ranks Australian areas into socio‐economic deciles according to several indexes, and is often used to explore associations between disadvantage and health outcomes [12]. Here, we used the Index of Relative Socioeconomic Disadvantage, a composite measure of relative disadvantage in an area derived from the weighted combination of a range of Census measures (e.g., income, education levels, employment status). Based on this, we categorised potential participants into ‘low’ (deciles 1 and 2), ‘medium’ (deciles 5 and 6) and ‘high’ (deciles 9 and 10) SES neighbourhood groups. From the eligible participant pool, we purposively sampled a similar number of participants from each group, while also ensuring a spread of ages, genders and drinking patterns (as measured through frequency of last month drinking and last month drinking of five or more drinks in an occasion) within each group. Participant demographics are provided in Table 1.

**TABLE 1:** Participant demographics.

Demographics	Number of participants
Age, years	
18–35	18
36–50	14
51+	13
Gender	
Man	18
Woman	24
Non‐binary	3
SEIFA group	
Low	16
Middle	17
High	12
Last month drinking	
Less often	0
At least monthly	2
At least weekly	13
A few times a week	21
Every day	9
Last month drinking 4+ drinks	
Less often	7
At least monthly	10
At least weekly	10
A few times a week	14
Every day	4

Abbreviation: SEIFA, socio‐economic indexes for areas.

Interviews were conducted by senior researchers with extensive interviewing experience Gabriel Caluzzi (man) and Amy Pennay (woman), and PhD students Klaudia Kepa (woman) and Alexandra Torney (woman) who received interview training for the project. Participants were first emailed with study information and given a verbal summary of the project from the interviewer. Written consent was gained prior to the interviews, which ranged from 25 min to 1 h. Most interviews were conducted via video call, although several were by phone or in‐person. Interviews followed a schedule exploring alcohol consumption and purchasing practices, alcohol‐related harms, attitudes towards alcohol and health more generally. Relevant questions for this study included ‘where do you buy most of your alcohol?’, ‘how much do you think you spend on alcohol each week?’ and ‘do you budget for alcohol?’. All interviews were recorded and transcribed externally. Ethics approval was granted by La Trobe University (Ethics ID HEC23088).

Our research question—how does neighbourhood socio‐economic status influence purchasing patterns?—was developed a priori as part of the larger project, although our analysis developed iteratively. The interviewers met and discussed early findings alongside data collection, noting interesting topics or themes. Once data collection was complete Gabriel Caluzzi, Klaudia Kepa and Amy Pennay developed a descriptive coding framework based off the data and interview topics. Gabriel Caluzzi, Klaudia Kepa and Amy Pennay coded the data using NVivo 12, and each coded interviews they did not conduct to familiarise themselves with the data and interview styles of the other researchers. This particular analysis drew heavily on the descriptive codes ‘purchasing patterns’ and ‘environmental and contextual factors’. We used constant comparison analysis [19] to check codes within the context of the original transcripts and compare data within and across groups, with a particular focus on differences and similarities in purchasing practices. Methodologically, we took a flexible grounded theory approach to the data [20], assuming knowledge as co‐constructed between researchers and participants, and focused on processes and actions that participants related to their alcohol purchasing. This informed the ecological perspective we applied to our data, and our application of thematic analysis [21], which involved multiple rounds of turning groups of codes into themes. As we refined the themes, we were reflexive in applying a theoretical framing that worked across our different public health and sociological disciplinary backgrounds, how coherent the themes were and how the final themes resonated with the interviewers.

Importantly, while we sampled based on neighbourhood SES, there was socio‐economic heterogeneity within the groups. Not all participants from socio‐economically disadvantaged neighbourhoods were socio‐economically disadvantaged themselves, and vice versa. Participants' reasons for living in an area included affordability, work/study location, proximity to family, and personal preference. Thus, relative socio‐economic disadvantage was used as an indication of local norms and environmental factors that might influence alcohol purchasing, rather than a proxy for individual SES.

## 3 RESULTS

Weekly alcohol expenditure ranged from approximately AU $10 to $300, with many participants reporting they spent around $40 to $50. This pattern was similar across the groups, although spending in the high SES neighbourhood group was more dispersed, with participants reporting spending both higher and lower amounts. Geographic contexts also varied; participants in low SES neighbourhoods typically lived near regional towns or in outer Melbourne suburbs, medium SES neighbourhood participants resided in Melbourne's middle‐outer suburbs and large regional centres, while high SES neighbourhood participants were closer to Melbourne's city centre. This distribution was an unintentional product of stratifying participants by SES neighbourhood, but aligns with Australia's general socio‐economic landscape [12].

The following sections explore five key interlinking themes, using individual accounts to represent broader patterns or underscore social norms. Pseudonyms are used for all participants, along with their gender, age, location (e.g., metropolitan) and SES neighbourhood group.

### 3.1 Accessibility of on‐premises venues

One key difference between SES neighbourhoods was how proximity to alcohol outlets shaped purchasing patterns. This was largely due to the travel time and physical accessibility of getting to venues. For participants in low SES neighbourhoods, attending on‐premises venues tended to be less common due to a need to drive rather than being able to walk or take public transport. As Isabella noted, this was particularly the case in regional areas:

‘I really think that most of the drinking in this area is just done privately in homes. Yeah, even—I mean, because another thing is that everybody drives here. It's a really sprawling area. The town of [regional city] itself is—it actually takes up a huge space considering the population. Yeah, everybody drives everywhere, so even out in restaurants and bars there are—quite a lot of people are actually drivers, so it's not really—it's not the same as being in a city where it's just—yeah, just this—it's a totally different culture. Yeah, you just take public transport everywhere […]’ (Isabella, woman, 34, low SES neighbourhood)

Comparing it with her past experiences in the city, Isabella suggested living regionally meant that venues were less accessible. This limited how much alcohol people purchased and consumed on‐premises, which in turn encouraged home drinking. Younger participants like Ayesha, who purchased alcohol on‐premises regularly, still had to be ‘conscious’ of their drinking due to limited transport options:

‘I think it really depends where you live. Like if I lived in the city and I could just hop on a train or walk to where I needed to go, that would probably influence my decision a lot. But I can't do that, so I do make conscious choices to not drink’. (Ayesha, woman, 26, low SES neighbourhood)

Many participants in medium SES neighbourhoods, primarily based in outer suburban areas, had greater access to bars, pubs and restaurants. Accordingly, a higher proportion of their purchasing tended to be done on‐premises—although purchasing could still be challenging for people like Lily who lived regionally.

‘In [regional city], there's no public transport. So, if I am going out—I went to a play reading last night, actually, and they had bubbles, but because I drove, I had half a glass of champagne, but I wasn't going to stick around to drink more. So, yeah, normally I'm either driving or I'm at home, drinking’. (Lily, woman, 42, medium SES neighbourhood)

The greatest contrast was with participants in the high SES neighbourhoods. While many in this group chose to purchase alcohol off‐premises, there were fewer barriers to purchasing on‐premises. Younger participants in particular, like Emily, described this accessibility:

‘It's [inner city suburb] probably the best place I've lived. It's super convenient. My apartment's only a few minutes' walk to a tram on one side and the train on the other. So it makes it really easy to get around and that kind of thing. Heaps of good cafes and restaurants and bars and everything’. (Emily, woman, 33, high SES neighbourhood)

Emily lived in the inner‐north side of the city and described the venues in close proximity to her. Because travel was less of a limiting factor in purchasing alcohol on‐premises, she attended licensed venues regularly.

### 3.2 Budgeting practices

Differences in budgeting for alcohol were also present. Across the sample, some participants acknowledged not budgeting for alcohol, particularly lighter drinkers and those who spent relatively little per week on alcohol. When participants did discuss budgeting, they often talked about setting price limits on specific beverages (usually bottles of wine) or spending on a night out. While it was difficult to separate out the effects of neighbourhood and individual‐level SES, there were some patterns of budgeting in the data. At times, local ecologies and accessibility played a clear role, as participants from low SES neighbourhoods often had to negotiate both the costs of drinking and the costs associated with getting back home.

‘I'm just mindful that for me a night out, because we live out of town too, that would involve a taxi home which would be another $50 on top of what I'm spending. Yeah, I just don't have that level of disposable income or that's not what I am choosing to spend my money on’. (Jasmine, woman, 48, low SES neighbourhood)

Even though Jasmine was not facing financial hardship, she found it difficult to justify the accumulated costs of a night out (‘I don't do it very often at all because I can't afford to’—Jasmine, woman, 48, low SES neighbourhood), preferring instead to buy alcohol to drink at home.

Many in medium SES neighbourhoods were generally price conscious too, often opting for off‐premises alcohol purchases or being selective about where they went out. For example, Alex described intentionality when going out (‘I am a little bit intentional about not just dropping a whole bunch of money on alcohol on a night out’—Alex, non‐binary, 36, medium SES neighbourhood) and Amanda talked about choosing affordable venues (‘I'll look at where we're potentially going because some places are more expensive than others’—Amanda, woman, 33, medium SES neighbourhood).

Similarly, Emily (high SES neighbourhood) talked about a variety of options for drinking venues, knowing which were cheaper, and had money she put aside for purchasing alcohol on‐premises:

‘I've got a set amount, which is just the discretionary fund money or whatever. So it's like, if I'm meeting friends or family or whatever, the dinner or a drink or something that I can spend that, and I don't worry about it’. (Emily, woman, 33, high SES neighbourhood)

There were also some patterns in terms of off‐premises purchasing. Some participants in the low SES neighbourhoods described limiting the amount they spent on a bottle (‘we buy bottles that are around about $15 usually’—Isabella, 34, low SES neighbourhood), but most did not talk about budgeting specifically for alcohol. For example, Garth described purchasing alcohol ‘with the money that I have left over from my groceries and stuff’ (Garth, man, 22, low SES neighbourhood) and Natalie said: ‘we just buy it as we need it, we don't give it [budgeting] a whole lot of thought’ (Natalie, woman, 48, low SES neighbourhood). Some of the low SES neighbourhood participants who did talk about setting a budget for themselves also felt they exceeded it, due in part to promotions:

‘I want to, like, $50 to $100, not more than that. But in the end, I'm spending like $300. So, I go a lot out of my budget, and when there's the offers, and these kind of things—yeah, so I grab a lot of things’. (Fatima, woman, 34, low SES neighbourhood).

Participants in the middle and high SES neighbourhood group often talked about setting limits on how much they would pay for a bottle of wine or carton of beer (‘I don't budget. I try to buy $30 bottle of wine’.—Lily, woman, 42, medium SES neighbourhood) or factoring it into their regular spending (‘I wouldn't go over $50—$60 in a week’—Monique, woman, 30, medium SES neighbourhood).

In general, participants from the middle and high SES neighbourhoods tended to talk about budgeting more in relation to a night out or a singular purchase of alcohol. This often resulted in having a general sense, or ‘ideal figure’ for their alcohol budget—even if they didn't always stick to it:

‘It's not guaranteed, but it's just more of an ideal figure that I want to stick to. It doesn't mean sometimes I stick to it, but yeah, case in point, this week it's been a little bit more. I know then other weeks, especially after Christmas and that, or more after the new year break it will wind down a little again’. (Matthew, man, 38, high SES neighbourhood)

Matthew was flexible with his alcohol spending and had a long‐term way of thinking about budgeting (i.e., not week‐to‐week). Similarly, while some high SES neighbourhood participants preferred not to spend money on‐premises, others acknowledged they could afford to do so without much concern for their budget:

‘I would never buy a bottle of beer in Dan Murphy's if it was $16 but I'm quite happy to spend $16 in the pub [unclear]. I mean, but I don't worry. If I'm going out for an evening, what it costs is what it costs. I guess I'm fortunate that I can afford to do that’. (Tom, 49, man, high SES neighbourhood)

Thus, while the high SES neighbourhood group described budgeting for alcohol, some also talked about making more expensive purchases and not being as concerned if they went over their usual budget.

### 3.3 Affordability

Closely connected with budgeting practices were more general discussions of affordability. These discussions centred around two main topics—navigating the rising cost of living and the affordability of wine. These topics resonated across all SES neighbourhood groups, indicating that financial concerns related to alcohol purchasing are a common consideration that might cut across neighbourhood‐level SES. Many participants talked about being more conscious about spending money on alcohol as they felt their regular expenses—bills, groceries etc.—had increased. This often led to reduced on‐premises drinking and socialising:

‘I think the cost of living has made it difficult to go out anyway, to socialise’. (Deborah, woman, 59, low SES neighbourhood)

‘The cost of living makes it interesting […]. That impacts how I drink sometimes too because obviously you factor in that it's just—there's a lot of cost involved, and you can't enjoy yourself as much as you want to’. (Matthew, man, 38, high SES neighbourhood)

Both Deborah and Matthew described consciously reining in their purchasing of alcohol on‐premises, substituting it with drinking at home to save money. One lighter drinker even talked about cutting out alcohol expenditure entirely (‘I think with the cost of living being what it is, if we were to have to cut something out that [alcohol] would probably be what we'd cut’—Megan, woman, 37, medium SES neighbourhood).

In contrast, many talked about the affordability of off‐premises purchasing. For a few, this seemed a conscious response to cost of living pressures. Although all alcohol was cheaper off‐premises, the availability of cheap wine was particularly important, with some substituting their preferred alcohol with wine to save money. For example, Sharon chose wine for its affordability:

‘Usually just wine, sometimes beer. It's just that wine was more affordable […] You can buy a bottle of wine for $5 or $6, so it's cheap and it's easy to drink at home’. (Sharon, woman, 59, low SES neighbourhood)

Similarly, some participants from other SES neighbourhoods intentionally substituted their preferred drink with wine because of the price (‘I switched to just wine because it's a lot cheaper’—Drew, non‐binary, 47, medium SES neighbourhood).

Although there was less talk of needing to substitute beverage types, participants in high SES neighbourhoods also talked about purchasing wine cheaply. They felt it was both affordable and good quality (‘I'm always blown away a bit that these days we can have such good wines cheaply’—Larry, man, 74, high SES neighbourhood) and how the price made it a desirable purchase (‘I look at the prices and go, I'll just buy my $4 bottle of wine’—Aoife, woman, 53, high SES neighbourhood).

While participants from high SES neighbourhoods acknowledged the affordability of wine, some were also more likely to describe alcohol as an affordable luxury:

‘If I'm buying good champagne, I'll pay for good champagne because good champagne's worth it, it's fabulous and good […] mixers are worth it. I'll buy Cointreau to make a margarita with because it's better than Triple Sec. I'll buy good tequila, I'll buy good gin …’ (Stan, man, 67, high SES neighbourhood)

While rising living costs limited some participants' alcohol purchases, others turned to cheaper off‐premises options like wine, which was seen as an affordable and desirable choice. Some high SES neighbourhood participants balanced affordability with the ability to indulge in higher‐end alcohol when they deemed it worth the price.

### 3.4 Purchasing motivations

Participants described three key motivations when purchasing alcohol—price, convenience and range. While these were relevant across all groups, the emphasis on each motivation varied between groups. For participants in the low SES neighbourhood group, the emphasis tended to be on convenience—although price, including specials and cheaper alternatives, was also important. Low SES neighbourhood participants often described purchasing from bottle shops nearby or co‐located next to supermarkets where they did regular shopping (‘mostly it's just convenience where I purchase from’—Jasmine, woman, 48, low SES neighbourhood). Two participants in this group also purchased alcohol online and had it delivered for price and convenience (‘Price, great offers, great deals—I always look out for that—and also you get to delivery at home, so it is more convenient’—Fatima, woman, 34, low SES neighbourhood). However, most noted it was not hard to buy alcohol as part of their regular grocery shopping.

The medium SES neighbourhood participants described a mix of convenience, price and range as factors, and tended to prefer larger warehousetype bottle stores when purchasing off‐premises (‘I'm going there for—because [it's] big, a lot of choices, discounts available and it's close to here’—Henry, man, 23, medium SES neighbourhood).

Price tended to be a key motivator for where and why participants in the high SES neighbourhood areas chose to purchase their alcohol off‐premises, as well as convenience (‘Price is what's driving me’—Stan, man, 67, high SES neighbourhood). Similar to the medium SES neighbourhood group, many preferred purchasing from larger bottle shops, as well as supermarkets that were licensed to sell alcohol (e.g., ALDI), although some also added that they could afford to not be selective:

‘I'll always go where it's the cheapest but if I'm buying a bottle of gin or if I'm buying good liquor, I pretty much don't care’. (Stan, man, 67, high SES neighbourhood)

In terms of on‐premises purchasing, commonly it was high SES neighbourhood participants that described particular motivators—perhaps because they had greater access to multiple venues. For example, some younger participants like Raj talked about having preferred bars and navigating drink specials around the city.

‘If there is a discount offer, some of my friends also have just that like, let's go to [bar] […] it's the offer and everything, so sometimes maybe because of the offer’. (Raj, man, 27, high SES neighbourhood)

Aoife, who had recently started working at a reduced salary, mentioned shifting to a venue with a happy hour to cut down on her spending:

‘I really just can't afford to go to my favourite bar […] but there's a place where you can sit and they have happy hour drinks, $5 glasses of wine. So, I go there because I can afford a $5 glass of wine’. (Aoife, woman, 53, high SES neighbourhood)

Thus, off‐premises, all groups prioritised convenience and price, with lower SES neighbourhood participants opting for nearby purchases, medium for range, and high for discounts (but with flexibility on premium buys). On‐premises, participants from the high SES neighbourhood had greater access to venues and were more likely to describe being motivated by preferences and drink specials.

### 3.5 Neighbourhood‐level norms

Neighbourhood‐level norms also influenced purchasing practices, largely through gentrification and the knock‐on effect this had for on‐ or off‐premises venues. In low SES neighbourhoods, particularly regional ones with fewer people and less nightlife, local venues often operated at reduced hours, limiting when and how often people could attend them:

‘… in terms of the pubs and bars, or what there is of bars. They're usually closed during the week’. (Isabella, woman, 34, low SES neighbourhood)

For others in low SES neighbourhoods, there were also some concerns around safety (‘there's a lot of drug and alcohol use within the area definitely, yeah, it's sometimes hard to go out and feel kind of safe’—Bianca, woman, 18, low SES neighbourhood). Cassidy, who identified as non‐binary, also described how they felt local venues were not safe spaces where they felt welcome (‘The local pub—someone who looks like me just gets ridiculed’—Cassidy, non‐binary, 31, low SES neighbourhood). This encouraged them to purchase alcohol off‐premises.

These discussions contrasted starkly with Emily's experience of living in a high SES neighbourhood where she regularly purchased alcohol on‐premises:

‘It's just a nice area, it's a really diverse mix of people, but everyone's really friendly and nice and it's—you feel safe here. You could go for a walk at 1:00AM down [busy inner‐city street] or whatever, and I don't think you'd have to worry too much about anything’. (Emily, woman, 33, high SES neighbourhood)

Emily's description highlights both the sense of safety, accessibility and opportunity she feels purchasing alcohol in her local inner‐city area.

Gentrification also came through in some discussions from the medium SES neighbourhood group. There was more talk of how off‐premises shops had changed over time. This included the introduction of large warehouse‐style bottle shops, which led some people to ‘stocking up’ (Carla, woman, 56, medium SES neighbourhood) when purchasing alcohol. Similarly, Lily described noticing a shift in the availability of what she considered good wine products:

‘The other thing that's happened here in [regional city], it's been really interesting to watch myself over the last year. So, […] there is a [grocery store] that sells amazing red wine, and the guy who runs the [grocery store], he sources it, and he sells it really cheaply, and it's always really good’. (Lily, woman, 42, medium SES neighbourhood)

These insights highlight how neighbourhood characteristics, shaped by gentrification in different ways, could influence participants' alcohol purchasing practices.

## 4 DISCUSSION

This study aimed to explore how neighbourhood SES influences alcohol purchase practices using an ecological approach. The three SES groups were quite heterogeneous, importantly highlighting that purchasing patterns are influenced by many factors beyond the local environment. Moreover, there were many similarities between the groups, as well as some notable differences. Unpacking these usefully allows us to think about how factors like geography, accessibility, and the affordability of alcohol influence purchasing patterns. It also highlights the importance of local alcohol policies that account for neighbourhood socio‐economic norms and infrastructure, while supporting broader policy measures that transcend SES differences.

Participants from different SES neighbourhoods described different degrees of physical access to on‐premises venues like bars and pubs. For many in low and some in medium SES neighbourhoods, proximity and lack of transport options could restrict purchasing drinks in venues. Concerns about drink‐driving, navigating public transport, costs associated with getting home and feelings of safety were all barriers. In contrast, those in high SES neighbourhoods, mostly the young participants, described purchasing alcohol in venues as accessible and often preferred. This highlights how local infrastructure can affect purchasing patterns. Barriers in lower SES areas may reinforce patterns of home drinking, potentially influencing consumption habits, social interaction and associated harms. Given home drinking has become a topic of greater research focus [22, 23], the link between access, purchasing and home drinking in different SES neighbourhoods may warrant further exploration. Additionally, preference for on‐premises drinking in higher SES neighbourhoods may reflect not just accessibility, but also distinct social practices and lifestyle choices within urban night‐time economies. Here, it may be useful to think of pragmatic policy options like regulating pricing and online sales that do not necessarily impact local hospitality businesses [24], as well as ways to enable equitable access to and from safe drinking environments via public transport infrastructure.

Budgeting for alcohol also differed across groups. While all participants were price conscious, those in low SES neighbourhoods described budgeting for alcohol as part of their general expenditure, while those in the middle and particularly high SES neighbourhoods tended to be more intentional in their budgeting. This may partly be because budgeting was often linked to on‐premises purchasing (i.e., not spending too much on a ‘night out’), which occurred more among high SES neighbourhood groups. In contrast, those from low SES neighbourhoods' purchasing off‐premises was more motivated by convenience—although money may have still been an underlying factor given the added financial and time costs involved with travelling further to buy alcohol. This suggests that factors such as accessibility and proximity to retail outlets may play a more significant role in their purchasing, as well as the wider infrastructure that enables or discourages travelling to particular alcohol outlets. It also suggests that limiting outlet density—a primary method for reducing alcohol consumption and related harms [4]—may have the greatest impact on low SES neighbourhood consumers, given the emphasis on accessibility among this group.

Although Australia has seen a long‐term rise in the price of alcohol relative to the cost of living over the past 30 years [25], cost of living concerns have increased after the COVID‐19 pandemic [26], which seemed to affect alcohol purchasing. Research from the UK suggests that, during recent cost of living pressures, cost motivated alcohol reduction among those less economically advantaged, but less so for other groups [27]. Here, it led to selecting cheaper venues, reducing outings, and switching to cheaper alternatives like wine across the neighbourhood SES groups. The affordability of wine was a particularly common theme, highlighting how Australia's currently inconsistent taxation of alcohol can influence beverage choices and purchasing in ways that might cut across SES. Currently, cheap wine attracts a lower tax burden and is relatively cheaper than beer and spirits to produce and purchase. This creates an incentive for producers to produce cheap wine that may need to be addressed in taxation policy. Minimum unit pricing, which sets a floor price for a standard drink of alcohol and restricts the sale of disproportionately cheap alcohol [28, 29], has been used to directly address the consumption of cheap wine in Australia [28, 30]. In line with the purchasing patterns and motives we saw in this study, it may have the potential to reduce the purchasing of cheap alcohol among consumers across SES groups. Increased prices could reduce the consumption and related harms associated with high‐volume, low‐cost alcohol. This is particularly important for heavier drinkers in lower SES groups, who purchase more alcohol at lower prices [16], but also experience higher levels of alcohol‐related mortality and morbidity than those who are more advantaged [31, 32].

There are some limitations to this study. Although we analysed patterns in the data according to neighbourhood SES, it was sometimes difficult to separate individual and neighbourhood‐level SES (e.g., patterns in budgeting and affordability). Neighbourhood SES should not be considered the same as individual SES; our aim has not been to focus on individual purchasing patterns, but rather on how local environments might shape purchasing. Thus, we may have missed elements of how class and individual SES shape alcohol purchasing. Moreover, factors such as age, gender and job/student status were important. For example, young people from all groups tended to talk more about drinking on‐premises, and women more often discussed safety concerns. While we touched on these factors, we were not able to explore them in detail in this study.

## 5 CONCLUSION

Our analysis of alcohol purchasing patterns across different SES neighbourhoods underscores how the local environment can influence purchasing practices. While regional differences, accessibility of on‐premises venues, and ways of budgeting for alcohol contributed to different purchasing patterns across SES neighbourhoods, this study highlights commonalities. Notably, the emphasis on off‐premises purchasing was a common theme, influenced by cost of living pressures and perceived affordability of alcohol options like wine. The findings emphasise the need for alcohol policy considerations that address community infrastructure and neighbourhood‐level socio‐economic norms, but also highlight the utility of some policy options that might cut across neighbourhood‐level SES.

## AUTHOR CONTRIBUTIONS

Each author certifies that their contribution to this work meets the standards of the International Committee of Medical Journal Editors.

## CONFLICT OF INTEREST STATEMENT

The authors declare no conflicts of interest.

## Data Availability

Research data are not shared.
